# Cervicothoracolumbar Multiregional Spinal Stenosis (MRSS): Outcomes of Single-Stage Surgery in a Case Series of 28 Patients With a Minimum Two-Year Follow-Up

**DOI:** 10.7759/cureus.111492

**Published:** 2026-06-25

**Authors:** Bharatkumar R Dave, Shivanand C Mayi, Sandesh Subhash Agrawal, Rushikesh B Shahade, Ajay Krishnan, Ravi Ranjan Rai, Mirant B Dave, Mikeson Panthackel, Arjit Vashishtha, Saurabh S Kulkarni, Yogenkumar Adodariya, Kishan Panjwani

**Affiliations:** 1 Spine Surgery, Stavya Spine Hospital and Research Institute, Ahmedabad, IND; 2 Spine Surgery, Shree Narayana Hospital, Raipur, IND; 3 Orthopedics, Sri Devaraj Urs Medical College, Sri Devaraj Urs Academy of Higher Education and Research, Kolar, IND; 4 Orthopedics and Traumatology, Shri Balaji Institute of Medical Science, Raipur, IND; 5 Orthopedics, Seth G.S. Medical College and King Edward Memorial Hospital, Mumbai, IND; 6 Spine Surgery, Bhavnagar Institute of Medical Science, Bhavnagar, IND; 7 Orthopedics, Mahatma Gandhi Medical College and Research Institute, Puducherry, IND

**Keywords:** cervico-dorso-lumbar stenosis, multiregional spinal stenosis, ossification of the ligamentum flavum (olf), single stage surgery, tandem stenosis

## Abstract

Background

Multiregional spinal stenosis (MRSS), defined as concomitant stenosis involving more than two nonconsecutive anatomically distinct spinal regions, represents a complex clinical entity with variable and often overlapping neurological manifestations. The coexistence of cervical, thoracic, and lumbar canal stenosis can obscure the primary symptomatic level, pose diagnostic challenges, and increase the risk of incomplete or staged management. Failure to recognize all affected regions may result in persistent symptoms and suboptimal functional recovery. This study aimed to characterize the clinicoradiological profile of cervicothoracolumbar MRSS and to evaluate the feasibility and perioperative parameters associated with single-stage surgical decompression.

Materials and methods

A retrospective cohort analysis was conducted on 28 consecutive patients who underwent single-stage surgical decompression for MRSS at a tertiary care center between January 2010 and March 2019, with a minimum follow-up duration of 24 months. Preoperative evaluation included a detailed neurological assessment and whole-spine MRI. Demographic characteristics, operative time (ORT), estimated blood loss (EBL), number of decompressed levels, surgical techniques employed, and intraoperative complications were recorded and analyzed.

Results

The study cohort comprised 28 patients (16 males and 12 females) with a mean age of 53.8 ± 9.6 years (range: 38-74 years). The mean ORT was 171.28 ± 48.13 minutes, and the mean EBL was 394.71 ± 131.14 mL. A total of 344 spinal levels were decompressed (mean: 12.28 levels per patient), involving the cervical (n = 123), thoracic (n = 151), and lumbar (n = 70) regions. Surgical procedures included anterior cervical decompression (n = 3), thoracic posterior instrumentation with pedicle screw fixation (n = 4), thoracic interbody fusion (n = 2), laminectomy (n = 9), and lumbar interbody fusion (n = 10). No major intraoperative complications were encountered.

Conclusions

MRSS should be strongly suspected in patients presenting with discordant, noncontiguous, or disproportionately severe neurological findings. Whole-spine MRI plays a pivotal role in identifying multiregional involvement and guiding comprehensive surgical planning. Single-stage decompression appears to be a safe and feasible strategy in carefully selected patients, demonstrating acceptable perioperative parameters.

## Introduction

Multiregional spinal stenosis (MRSS) is defined as the presence of spinal canal narrowing involving more than two anatomically distinct regions of the spine, including the cervical, thoracic, and lumbar segments. It represents a complex and increasingly recognized clinical entity in contemporary spine practice. While tandem spinal stenosis (TSS), traditionally describing involvement of two regions, has been widely reported, MRSS provides a broader and more inclusive framework encompassing multiregional and, in rare cases, triple-region disease [1,2].

The overall prevalence of spinal stenosis in the general population is reported to range between 11% and 39%, with higher rates observed in elderly individuals due to progressive degenerative changes. TSS has been reported in approximately 5-25% of patients with spinal stenosis, depending on the population studied and the diagnostic criteria used [3-5]. However, the true incidence and prevalence of MRSS, particularly involving all three regions of the spine, remain poorly defined, largely due to underdiagnosis and variability in clinical presentation.

Although multilevel degenerative changes are common in the aging population, simultaneous symptomatic involvement of multiple spinal regions remains underrecognized and may present with overlapping or discordant neurological findings [6]. This diagnostic complexity increases the risk of incomplete decompression if all contributing levels are not accurately identified. The clinical scenario is further complicated by associated pathologies such as intervertebral disc herniation, ossification of the ligamentum flavum, ossification of the posterior longitudinal ligament, and metabolic conditions such as fluorosis, all of which may contribute to multilevel spinal canal compromise [4].

Previous studies have demonstrated that following decompression at a single symptomatic level, previously asymptomatic stenotic segments in other regions may become clinically significant, resulting in new or progressive neurological deficits and suboptimal functional outcomes [6]. Furthermore, unrecognized stenosis in nonoperated regions, particularly in the thoracic spine, has been associated with delayed neurological deterioration after initial surgery, highlighting the importance of comprehensive preoperative evaluation of the entire spine [7]. This deterioration may be partly explained by persistent multilevel cord compression and impaired spinal cord perfusion, including venous congestion, although direct evidence in the context of multiregional stenosis remains limited.

Traditionally, the management of MRSS has involved staged surgical procedures, prioritizing the most symptomatic or neurologically dominant level [8]. However, emerging evidence suggests that single-stage decompression across multiple spinal regions may offer several advantages, including improved neurological recovery, reduced cumulative surgical morbidity, shorter overall hospitalization, and enhanced patient satisfaction [9,10]. Favorable outcomes following single-stage or combined decompressive procedures have been reported in selected patient populations, supporting its role as an effective and feasible treatment strategy [11].

Tandem stenosis refers to clinically significant spinal canal narrowing involving two noncontiguous regions, whereas MRSS denotes radiological multilevel stenosis with clinical correlation. This distinction is important when interpreting imaging findings and planning surgical management. Involvement of all three spinal regions, such as cervical, thoracic, and lumbar, remains rare, with only a limited number of reports describing cervicothoracolumbar stenosis and its management using either staged or single-stage surgical approaches [12,13]. Consequently, there is a paucity of evidence regarding optimal surgical strategies, perioperative considerations, and long-term outcomes in this subset of patients.

In this study, we present a case series of 28 patients with MRSS involving two or more noncontiguous spinal regions who underwent single-stage surgical decompression with a minimum follow-up of five years. The surgical approach primarily involved posterior decompression, with or without stabilization, depending on the underlying pathology and intraoperative findings. This study aims to evaluate the clinicoradiological characteristics, surgical considerations, and perioperative outcomes associated with this underreported condition. With increasing life expectancy and the widespread use of whole-spine MRI, the recognition of MRSS is expected to increase, emphasizing the need for improved diagnostic awareness and standardized management strategies.

## Materials and methods

Study design and patient selection

This retrospective observational study was conducted at Stavya Spine Hospital and Research Institute, Ahmedabad, India. Institutional Ethics Committee approval was obtained before the study (protocol no.: SSHRI/CS/NS/MRSS/BRD/107/04.26). Patients who underwent surgery for MRSS between January 2010 and March 2019 were included if they had at least 48 months of follow-up. Demographic data, including age, sex, and duration of symptoms, were obtained from medical records. A history of trivial trauma associated with acute worsening of preexisting symptoms was also documented.

Patients were included in the study if they demonstrated radiological evidence of cervical, thoracic, and lumbar spinal stenosis on whole-spine MRI, along with appropriate clinicoradiological correlation. Only patients with symptoms of myelopathy were included. Only those patients who were deemed medically fit to undergo single-stage surgery were considered eligible for inclusion. In addition, all participants were required to provide informed consent for both the surgical procedure and the postoperative rehabilitation protocol before enrollment in the study.

Patients were excluded from the study if they were unwilling to provide consent for participation, surgery, or postoperative rehabilitation. Individuals younger than 18 years of age were also excluded. Furthermore, patients with a previous history of spinal surgery were not considered eligible for inclusion in the study.

Clinical and radiological evaluation

All patients underwent a comprehensive clinical and neurological evaluation. Functional status was assessed using validated scoring systems, including Nurick grade [14], the Oswestry Disability Index (ODI) [15], and the modified Japanese Orthopaedic Association (mJOA) score [16]. Radiological evaluation comprised plain radiographs and whole-spine MRI to assess the extent and severity of spinal stenosis. Cervical and thoracic stenosis were graded according to Kang et al. [17], while lumbar stenosis was graded using the Schizas classification based on axial T2-weighted images [18].

Surgical decision-making was guided by clinicoradiological correlation. Cervical decompression was considered in patients with clinical features of myelopathy and corresponding imaging findings. Thoracic or lumbar decompression was performed in patients presenting with lower-limb symptoms such as neurogenic claudication or radiculopathy, supported by radiological evidence of stenosis.

Surgical procedure

All procedures were performed under general anesthesia. Single-stage decompression of the cervical, thoracic, and lumbar regions was carried out using a dual-team, two-site simultaneous surgical approach, allowing parallel operative work at different spinal regions to optimize operative efficiency. An ultrasonic bone scalpel was utilized for bony decompression across all regions to facilitate precise bone removal while minimizing neural and soft-tissue injury.

In patients with significant anterior cervical pathology, anterior cervical decompression and fusion were performed before posterior procedures. Posterior decompression with or without instrumentation was performed depending on the extent of disease and spinal stability. Cervical procedures included laminectomy with or without cervical pedicle screw or lateral mass screw fixation; thoracic procedures included laminectomy with pedicle screw fixation when required; and lumbar procedures included decompression with or without fusion using transforaminal lumbar interbody fusion or posterolateral fusion in cases with instability. Operative time (ORT), estimated blood loss (EBL), number of levels operated on, and intraoperative complications were recorded.

Postoperative care and outcome assessment

Patients were mobilized early with supervised physiotherapy starting on the second postoperative day. Deep vein thrombosis prophylaxis was administered to high-risk patients according to NICE guidelines [19]. Patients were typically discharged on postoperative days 5-6. Patients were followed up at six weeks, three months, six months, and one year, with additional visits scheduled as necessary based on clinical progress.

Outcome assessment included systematic evaluation of neurological and functional status using Nurick grade, the mJOA score, and the ODI. Perioperative complications, such as neurological worsening, dural injury, and pneumocephalus, were documented at each follow-up to provide a comprehensive overview of both recovery and adverse events.

Statistical analysis

Statistical analysis used descriptive and comparative methods. Continuous variables were expressed as mean ± SD and range (minimum-maximum). Categorical variables were presented as frequencies and percentages. Preoperative and postoperative clinical outcome scores, including Nurick grade, mJOA score, and ODI, were compared where applicable. Data were assessed for normality, and appropriate statistical tests were applied. For normally distributed variables, paired t-tests were used, while the Wilcoxon signed-rank test was used for nonnormally distributed data. A p-value of <0.05 was considered statistically significant.

## Results

A total of 28 patients (16 males and 12 females) were included in the study, with a mean age of 53.8 years (range: 38-74 years). The mean follow-up duration was 38.76 ± 22.17 months (range: 24-108 months). The mean duration of symptoms before presentation was 26.8 ± 6.5 months (range: 1-180 months). The mean duration of cervical and lumbar symptoms before surgery was 4.6 ± 2.4 months and 5.6 ± 1.4 months, respectively. The mean ORT was 171.28 ± 48.13 minutes (range: 90-270 minutes). The mean EBL was 394.71 ± 131.14 mL (range: 130-750 mL), and the mean number of blood transfusion units required was 0.81 ± 1.00 (range: 1-4 units), as demonstrated in Table 1.

**Table 1:** Parameters and mean values EBL, estimated blood loss; ORT, operative time

Parameter	Value
Total patients	28
Male	16/28 (57.14%)
Female	12/28 (42.85%)
Mean age (years)	53.8 ± 9.0 (38-74)
Follow-up (months)	38.76 ± 22.17 (24-108)
Duration of symptoms (months)	26.8 ± 6.5 (1-180)
Cervical symptoms (months)	4.6 ± 2.4
Lumbar symptoms (months)	5.6 ± 1.4
ORT (minutes)	171.28 ± 48.13 (90-270)
Blood transfusion (units)	0.81 ± 1.00 (range: 0-4 units)
EBL (mL)	394.71 ± 131.14 (130-750)

Extent of decompression and surgical details

A total of 344 spinal levels were decompressed, with a mean of 12.28 levels per patient. Of these, 123 levels were cervical, 151 were thoracic, and 70 were lumbar (Table 2). Surgical procedures included anterior cervical decompression (n = 3), thoracic pedicle screw fixation (n = 4), thoracic interbody fusion (n = 2), laminectomy (n = 9), and lumbar interbody fusion (n = 10) (Table 3).

**Table 2:** Distribution of operated levels

Region	Total levels	Mean levels per patient
Cervical	123	4.39
Thoracic (dorsal)	151	5.39
Lumbar	70	2.5
Total	344	12.28

**Table 3:** Types of surgical procedures

Surgical procedure	Number of patients
Anterior cervical decompression	3 (10.71%)
Thoracic pedicle screw fixation	4 (14.28%)
Thoracic interbody fusion	2 (7.14%)
Lumbar interbody fusion	10 (35.71%)
Laminectomy	9 (32.14%)
Total	28 (100%)

Clinical outcomes

The mean mJOA score improved from 8.86 ± 3.06 preoperatively to 13.00 ± 2.26 at 12 months, corresponding to a mean improvement of 4.1 points (Table 4). The mean mJOA recovery rate was 48.23 ± 26.90%, indicating moderate neurological recovery. Similarly, the mean Nurick grade improved from 3.83 ± 0.87 preoperatively to 2.23 ± 1.16 at 12 months and further improved to 1.96 ± 1.17 at final follow-up, suggesting sustained functional recovery over time (Table 4). The ODI also demonstrated significant improvement, decreasing from a mean preoperative value of 68.15 ± 22.77 to 30.11 ± 16.27 at 12 months, reflecting a marked reduction in disability. Overall, all clinical outcome measures demonstrated significant improvement following single-stage multiregional decompression.

**Table 4:** Clinical outcomes mJOA, modified Japanese Orthopaedic Association; ODI, Oswestry Disability Index

Parameter	Preoperative	Postoperative (12 months)	Improvement
mJOA score	8.86 ± 3.06	13.00 ± 2.26	4.1
Nurick grade	3.83 ± 0.87	2.23 ± 1.16	1.6-point improvement
ODI	68.15 ± 22.77	30.11 ± 16.27	38-point reduction

Complications

**Figure 1:**
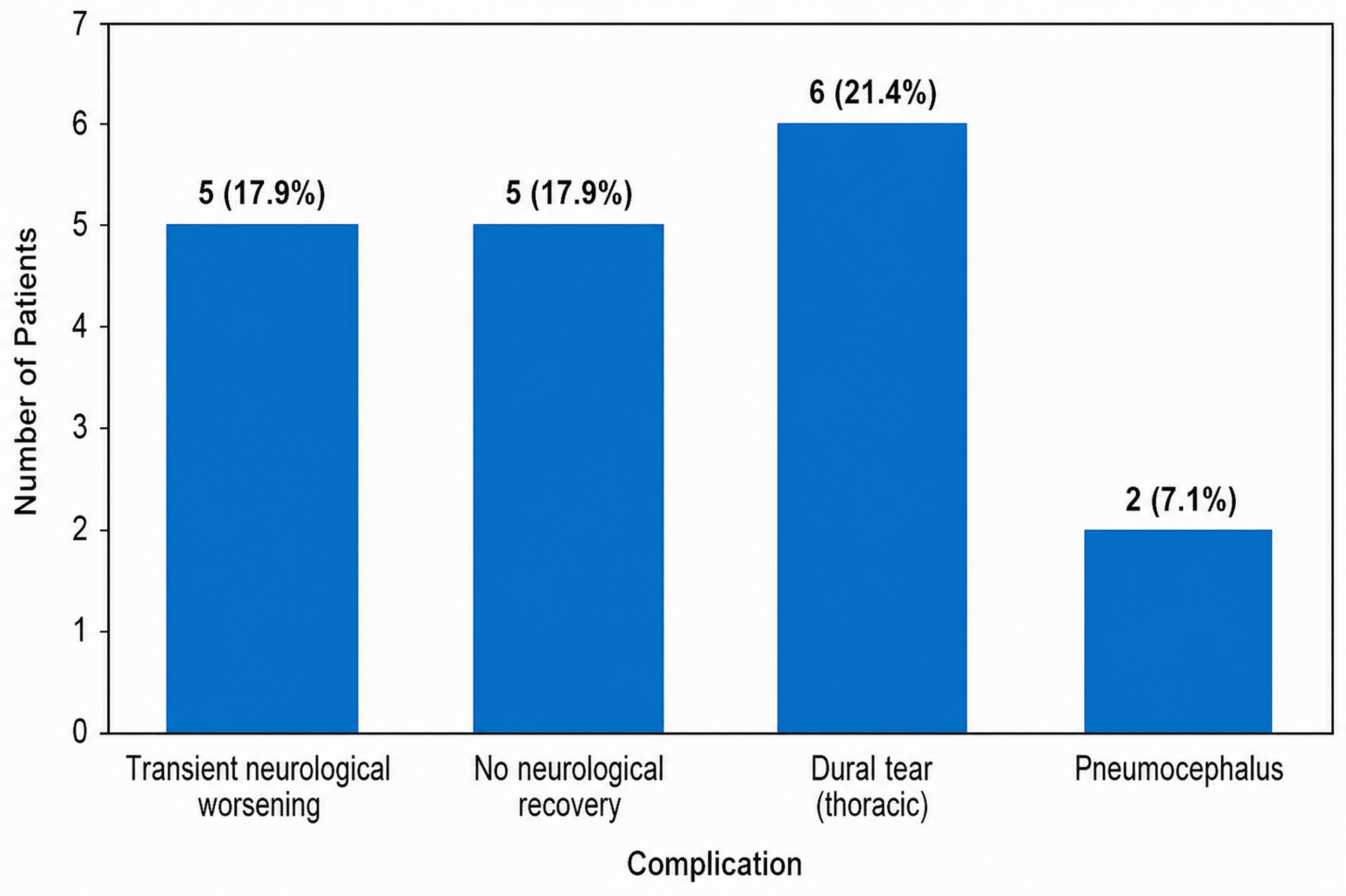
Distribution of perioperative complications

Perioperative complications were observed in a subset of patients. Transient neurological worsening was noted in five patients, while five patients showed no neurological recovery at final follow-up. Dural tears were encountered in six cases, all involving the thoracic spine, and were managed intraoperatively without long-term sequelae. Two patients developed symptomatic pneumocephalus. The distribution of complications is shown in Figure 1. No major life-threatening complications or mortality were observed in the study cohort.

## Discussion

MRSS involving the cervical, thoracic, and lumbar spine represents a rare and complex clinical entity characterized by overlapping neurological manifestations and diagnostic challenges [17,18]. The literature specifically addressing true multiregional (cervical, thoracic, and lumbar) spinal stenosis is sparse. Hong and Liu reported a rare case of MRSS involving all three spinal regions, managed with staged procedures due to the complexity of the pathology; however, the patient experienced complications, including epidural hematoma and interval neurological deterioration between surgeries [20]. In contrast, Schaffer et al. described successful management of triple-level spinal stenosis with single-stage decompression, achieving favorable clinical outcomes and thereby highlighting the feasibility of this approach in carefully selected patients [21]. These contrasting reports emphasize the need for individualized surgical planning based on patient-specific factors and disease characteristics.

The phenomenon of concurrent multilevel stenosis was first described by Teng and Papatheodorou in 1964 [22,23] and was later termed “TSS” by Dagi et al., who characterized it by a classical triad of intermittent neurogenic claudication, progressive gait disturbance, and combined upper and lower motor neuron involvement affecting both the upper and lower extremities. Multiregional stenosis often presents with overlapping symptoms, leading to diagnostic uncertainty. TSS, typically involving the cervical and lumbar spine, is reported in 5-25% of patients, highlighting that multilevel involvement is not uncommon [24,25].

The clinical complexity of MRSS arises from the coexistence of upper motor neuron and lower motor neuron signs, along with myelopathic and radicular symptoms, which often leads to significant clinicoradiological discordance [7,12]. This discordance is further compounded by age-related degenerative changes such as spondylosis, ligamentum flavum hypertrophy, and intervertebral disc degeneration. Additional pathologies, including ossified ligamentum flavum and ossified posterior longitudinal ligament, further contribute to multilevel neural compression [26]. Additionally, the presence of multiple compressive lesions may mask classical neurological findings; for example, exaggerated deep tendon reflexes in the lower limbs due to cervical myelopathy may be attenuated or obscured in the presence of concomitant lumbar stenosis, thereby making localization of the primary symptomatic level particularly challenging [3]. In our study cohort, all patients presented with gait disturbances and clinical features suggestive of myelopathy involving both the upper and lower extremities, underscoring the complexity and severity of multiregional involvement. Unlike TSS, which typically involves two regions, MRSS encompasses more extensive multiregional involvement, including triple-region disease as observed in the present study.

The widespread use of whole-spine MRI has improved the detection of multiregional stenosis, revealing that a significant proportion of patients with symptomatic disease at one level may harbor additional lesions at other levels. However, the clinical relevance of these findings remains variable, emphasizing the importance of clinicoradiological correlation [26,27]. Park et al. demonstrated that approximately 12.1% of patients with symptomatic lumbar stenosis had concomitant cervical or thoracic stenosis on imaging, while Matsumoto et al. reported that up to 78.7% of asymptomatic individuals had radiological evidence of degenerative changes in both the cervical and lumbar regions [27-29]. Despite these findings, the true clinical incidence of cervicothoracolumbar MRSS remains poorly defined, and there is currently no universally accepted classification system or standardized treatment protocol for this complex condition, reflecting a significant gap in the existing literature.

Traditionally, several authors have advocated addressing cervical stenosis first, particularly in patients with myelopathy, as lower-extremity symptoms may be partially attributable to cervical cord compression and may improve following cervical decompression. Conversely, other authors have proposed that the region with the most severe or functionally limiting symptoms should be prioritized to achieve maximal immediate clinical benefit [30,31]. However, staged surgical interventions, although potentially safer in selected high-risk patients, have several limitations. These include cumulative anesthetic exposure, prolonged treatment duration, increased health care costs, and the risk of interval neurological deterioration [9,11].

The optimal surgical strategy for MRSS remains controversial. Despite the overall favorable outcomes observed in our series, certain complications were encountered, including transient neurological worsening, dural tears, and pneumocephalus. Extensive multilevel decompression is associated with an increased risk of dural injury and CSF leakage. Pneumocephalus, defined as the presence of air within the cranial cavity, is a rare but recognized complication following spinal surgery, most commonly associated with dural breach and CSF loss [32,33]. The underlying mechanism is thought to involve a ball-valve effect, allowing intracranial air entry in the setting of reduced intracranial pressure due to CSF leakage. Clinically, patients may present with headache, nausea, altered sensorium, or delayed neurological recovery. Although most cases can be managed conservatively, delayed or tension pneumocephalus may require urgent intervention.

In contrast, single-stage simultaneous decompression has gained increasing acceptance as a viable treatment strategy, offering several advantages, including reduced overall hospitalization time, avoidance of interval neurological deterioration, and improved patient satisfaction due to a single definitive intervention [8,10]. Multiple studies have demonstrated favorable outcomes with single-stage decompression in patients with tandem stenosis, with acceptable complication rates and significant functional improvement [13]. In our study, single-stage decompression across all three regions was feasible, with acceptable complication rates and satisfactory neurological improvement at two-year follow-up. These findings support the safety and efficacy of a single-stage approach when clinically and radiologically indicated.

In the present study, all patients underwent single-stage decompression of the cervical, thoracic, and lumbar regions, allowing us to comprehensively address all symptomatic levels in a single operative session. A key strength of our study was the use of a coordinated two-team approach, enabling simultaneous decompression across spinal regions. This strategy significantly improved operative efficiency, reduced surgical time, and minimized intraoperative blood loss. The use of an ultrasonic bone scalpel has been shown to facilitate rapid and safe multilevel decompression, with a reduced risk of dural injury and neural tissue damage, which was consistent with our intraoperative experience [31]. The present study demonstrates that single-stage decompression for cervicothoracolumbar MRSS is a feasible and effective approach.

Socioeconomic factors also influenced treatment decisions, as many patients presented with advanced disease and preferred a single definitive procedure due to financial and logistical constraints. This highlights the practical relevance of single-stage surgery in resource-constrained settings, where multiple staged procedures may not be feasible or acceptable to patients [27-30]. Despite the overall favorable outcomes observed in our series, certain complications were encountered, including transient neurological worsening, dural tears, and pneumocephalus, which are known complications associated with extensive multilevel decompressive procedures [31]. Additionally, a subset of patients demonstrated delayed neurological deterioration during follow-up, which may be attributed to the progressive nature of degenerative spinal disease at untreated levels or incomplete neural recovery.

This study has several limitations, including its retrospective design and relatively small sample size, which may limit generalizability. Additionally, long-term biomechanical outcomes and spinal alignment were not assessed. Future prospective studies with larger cohorts and standardized outcome measures are required to validate these findings and establish evidence-based guidelines. Despite these limitations, this study provides valuable insights into the management of this rare and complex condition and supports the role of single-stage surgery as a viable treatment strategy.

## Conclusions

Cervicothoracolumbar MRSS is an increasingly recognized clinical entity characterized by complex and overlapping neurological presentations, often leading to diagnostic challenges and delayed management. A high index of suspicion, combined with comprehensive neurological evaluation and whole-spine MRI, is essential to achieve accurate clinicoradiological correlation and avoid missed or untreated stenotic segments.

Single-stage decompression addressing all involved regions appears to be a feasible and effective surgical strategy in appropriately selected patients, providing satisfactory functional recovery with acceptable perioperative risk. In our series, selection criteria for single-stage surgery included radiological evidence of significant stenosis at the cervical, thoracic, and lumbar levels on whole-spine MRI; clinical correlation between neurological deficits and the identified levels of compression; absence of contraindicating comorbidities that would preclude prolonged anesthesia; and patient consent to undergo a comprehensive surgical and rehabilitation plan. Additional factors influencing patient selection were overall functional status, the severity and progression of neurological symptoms, and the patient's ability to participate in postoperative rehabilitation. Careful patient selection, meticulous preoperative planning, coordinated surgical execution, and structured postoperative rehabilitation are critical factors influencing successful outcomes.

## References

[1] Epstein NE, Epstein JA, Carras R, Murthy VS, Hyman RA (1984). Coexisting cervical and lumbar spinal stenosis: diagnosis and management. Neurosurgery.

[2] Caron TH, Bell GR (2007). Combined (tandem) lumbar and cervical stenosis. Semin Spine Surg.

[3] van Eck CF, Spina Iii NT, Lee JY (2017). A novel MRI classification system for congenital functional lumbar spinal stenosis predicts the risk for tandem cervical spinal stenosis. Eur Spine J.

[4] Li WJ, Guo SG, Sun ZJ, Zhao Y (2015). Multilevel thoracic ossification of ligamentum flavum coexisted with/without lumbar spinal stenosis: staged surgical strategy and clinical outcomes. BMC Musculoskelet Disord.

[5] Luo CA, Kaliya-Perumal AK, Lu ML, Chen LH, Chen WJ, Niu CC (2019). Staged surgery for tandem cervical and lumbar spinal stenosis: which should be treated first?. Eur Spine J.

[6] Swanson BT (2012). Tandem spinal stenosis: a case of stenotic cauda equina syndrome following cervical decompression and fusion for spondylotic cervical myelopathy. J Man Manip Ther.

[7] Fushimi K, Miyamoto K, Hioki A, Hosoe H, Takeuchi A, Shimizu K (2013). Neurological deterioration due to missed thoracic spinal stenosis after decompressive lumbar surgery: a report of six cases of tandem thoracic and lumbar spinal stenosis. Bone Joint J.

[8] Gupta A, Dave B, Nanda A, Modi H (2009). Concomitant noncontiguous level (thoracic & lumbar) spinal stenosis. Int Orthop.

[9] Eskander MS, Aubin ME, Drew JM (2011). Is there a difference between simultaneous or staged decompressions for combined cervical and lumbar stenosis?. J Spinal Disord Tech.

[10] Kikuike K, Miyamoto K, Hosoe H, Shimizu K (2009). One-staged combined cervical and lumbar decompression for patients with tandem spinal stenosis on cervical and lumbar spine: analyses of clinical outcomes with minimum 3 years follow-up. J Spinal Disord Tech.

[11] Chen Y, Chen DY, Wang XW, Lu XH, Yang HS, Miao JH (2012). Single-stage combined decompression for patients with tandem ossification in the cervical and thoracic spine. Arch Orthop Trauma Surg.

[12] Molinari RW, Flanigan R, Yaseen Z (2012). Tandem spinal stenosis (TSS): literature review and report of patients treated with simultaneous decompression. Curr Orthop Pract.

[13] Krishnan A, Dave BR, Kambar AK, Ram H (2014). Coexisting lumbar and cervical stenosis (tandem spinal stenosis): an infrequent presentation. Retrospective analysis of single-stage surgery (53 cases). Eur Spine J.

[14] Nurick S (1972). The pathogenesis of the spinal cord disorder associated with cervical spondylosis. Brain.

[15] Fairbank JC, Couper J, Davies JB, O'Brien JP (1980). The Oswestry low back pain disability questionnaire. Physiotherapy.

[16] Benzel EC, Lancon J, Kesterson L, Hadden T (1991). Cervical laminectomy and dentate ligament section for cervical spondylotic myelopathy. J Spinal Disord.

[17] Kang Y, Lee JW, Koh YH, Hur S, Kim SJ, Chai JW, Kang HS (2011). New MRI grading system for the cervical canal stenosis. AJR Am J Roentgenol.

[18] Schizas C, Theumann N, Burn A, Tansey R, Wardlaw D, Smith FW, Kulik G (2010). Qualitative grading of severity of lumbar spinal stenosis based on the morphology of the dural sac on magnetic resonance images. Spine (Phila Pa 1976).

[19] Venous thromboembolism in over 16s: reducing the risk of hospital-acquired deep vein thrombosis or pulmonary embolism (2018). Venous thromboembolism in over 16s: reducing the risk of hospital-acquired deep vein thrombosis or pulmonary embolism. NICE.

[20] Hong CC, Liu KP (2015). A rare case of multiregional spinal stenosis: clinical description, surgical complication, and management concept review. Global Spine J.

[21] Schaffer JC, Raudenbush BL, Molinari C, Molinari RW (2015). Symptomatic triple-region spinal stenosis treated with simultaneous surgery: case report and review of the literature. Global Spine J.

[22] Hill J, Treasure T (2007). Reducing the risk of venous thromboembolism (deep vein thrombosis and pulmonary embolism) in inpatients having surgery: summary of NICE guidance. BMJ.

[23] Teng P, Papatheodorou C (1964). Combined cervical and lumbar spondylosis. Arch Neurol.

[24] Dagi TF, Tarkington MA, Leech JJ (1987). Tandem lumbar and cervical spinal stenosis. Natural history, prognostic indices, and results after surgical decompression. J Neurosurg.

[25] Nagata K, Yoshimura N, Hashizume H (2017). The prevalence of tandem spinal stenosis and its characteristics in a population-based MRI study: the Wakayama Spine Study. Eur Spine J.

[26] Hou X, Sun C, Liu X (2016). Clinical features of thoracic spinal stenosis-associated myelopathy: a retrospective analysis of 427 cases. Clin Spine Surg.

[27] Aydogan M, Ozturk C, Mirzanli C, Karatoprak O, Tezer M, Hamzaoglu A (2007). Treatment approach in tandem (concurrent) cervical and lumbar spinal stenosis. Acta Orthop Belg.

[28] Park MS, Moon SH, Kim TH, Oh JK, Lyu HD, Lee JH, Riew KD (2015). Asymptomatic stenosis in the cervical and thoracic spines of patients with symptomatic lumbar stenosis. Global Spine J.

[29] Matsumoto M, Okada E, Toyama Y, Fujiwara H, Momoshima S, Takahata T (2013). Tandem age-related lumbar and cervical intervertebral disc changes in asymptomatic subjects. Eur Spine J.

[30] Yamada T, Yoshii T, Yamamoto N (2018). Clinical outcomes of cervical spinal surgery for cervical myelopathic patients with coexisting lumbar spinal canal stenosis (tandem spinal stenosis): a retrospective analysis of 297 cases. Spine (Phila Pa 1976).

[31] Dave BR, Degulmadi D, Dahibhate S, Krishnan A, Patel D (2019). Ultrasonic bone scalpel: utility in cervical corpectomy. A technical note. Eur Spine J.

[32] Dave BR, Jain A, Degulmadi D, Krishnan A, Bang P (2020). Symptomatic pneumocephalus following spine surgery: an institutional experience and review of literature. Indian Spine J.

[33] Lim Y, Dahapute A, Clarke A, Hutton M, Selbi W (2024). Delayed tension pneumocephalus and pneumorrhacis after routine cervical spine surgery treated successfully without burr holes. Ann R Coll Surg Engl.

